# One-for-one or one-for-all? Considerations for filovirus vaccine development

**DOI:** 10.1371/journal.pbio.3003142

**Published:** 2025-04-14

**Authors:** Andrea Marzi

**Affiliations:** Laboratory of Virology, Division of Intramural Research, National Institute of Allergy and Infectious Diseases, National Institutes of Health, Hamilton, Montana, United States of America

## Abstract

Filoviruses continue to re-emerge in Africa, causing localized public health emergencies. Vaccination has slowly been implemented for Ebola virus, but not other filoviruses. This Perspective highlights the importance of taking a holistic approach to protect against filoviruses, discussing possible strategies to achieve cross-protection.

## Teaser

Filoviruses continue to re-emerge in Africa, causing localized public health emergencies. Although vaccination has slowly been implemented for Ebola virus, not for other filoviruses, holistic approaches are needed to broadly protect against filovirus threats.

Almost six decades ago, filoviruses were identified as the causative agents of hemorrhagic disease outbreaks in humans. Despite the fact that these diseases have high fatality rates, only countermeasures against one filovirus, Ebola virus (EBOV), have been approved to date [1]. Filovirus outbreaks are thought to originate through spillover from bats, which have been suggested as the filovirus reservoir, to intermediate hosts or end hosts like humans, mostly in remote locations in Africa [2]. Although this occurs infrequently and most outbreaks are small, large outbreaks were reported in 2014 and 2020. From December 2013 until 2016, EBOV devastated the fragile healthcare infrastructure in Guinea, Liberia and Sierra Leone with over 28,000 cases and over 11,000 fatalities [3]. Single cases were imported to other countries, including Nigeria, Mali, Italy, Spain, the United Kingdom and the United States of America (USA); in the USA EBOV transmission occurred from the imported case to healthcare workers [3, 4]. In 2018–2020, the Democratic Republic of the Congo (DRC) reported its 10th—and, to date, largest—EBOV outbreak, with 3,470 cases and 2,287 fatalities [3]. Since 2021, the filoviruses Sudan virus (SUDV) and Marburg virus (MARV) have also garnered global attention, causing outbreaks of hemorrhagic disease: SUDV in Uganda (2022, 2025) [3], and MARV in Guinea (2021), Ghana (2022), Equatorial Guinea (2023), Tanzania (2023) and Rwanda (2024) [5]. MARV is particularly concerning, as its most recent spillovers suggest the expansion of its endemic area into regions where other filoviruses, like EBOV, SUDV and Bundibugyo virus (BDBV), are already endemic. The 2014–2016 EBOV epidemic highlighted another underrecognized aspect of filovirus disease—viral persistence and recrudescence. Growing evidence shows that EBOV and MARV can be sexually transmitted by disease survivors [2]. Vaccination of sexual partners of survivors or treatment of the survivors themselves should therefore be considered. Together, these issues highlight the need for deployable countermeasures and mitigation strategies to temper future outbreaks. Where do we stand today?

**Fig 1:**
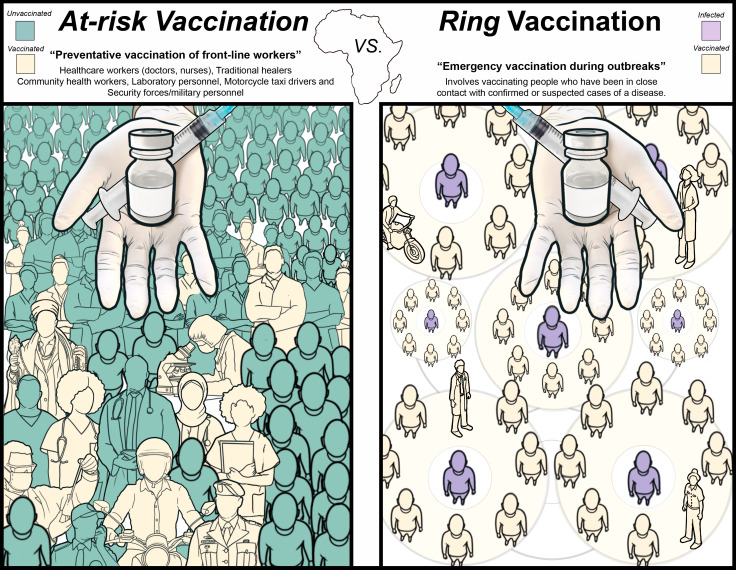
At-risk vaccination vs. ring-vaccination. Strategies to combat infrequent infectious disease outbreaks with high public health impact.

Filovirus vaccine development has traditionally been focused on specific vaccines for each virus. Before the 2014–2016 West African EBOV epidemic, there were no licensed vaccines or treatments for this disease at all; although several experimental approaches had shown uniform protection in the gold-standard nonhuman primate (NHP) model [6], human vaccine trials had rarely been conducted [6]. The 2014–2016 epidemic accelerated EBOV countermeasure development and, together with trials conducted during the 2018–2020 outbreak in the DRC [2], resulted in two vaccines and two monoclonal antibody therapies approved for human use today [1]. The first approved vaccine was a fast-acting, live-attenuated vaccine based on vesicular stomatitis virus (VSV) that was shown to elicit protective responses within 10 days—this is ideal for outbreak response [7]. The second vaccine consisted of an adenovirus 26 (Ad26-EBOV) initial dose, known as prime, followed by a heterologous second dose (boost) with modified Vaccinia Ankara virus (MVA-BN-filo)—a strategy well-suited for mass vaccination [8]. Yet, only in 2022 did African countries where EBOV is endemic start preventive vaccination of the at-risk populations [9] (Fig 1). In 2024, the Global Alliance for Vaccines and Immunization (GAVI) announced a campaign to facilitate preventive EBOV vaccination for at-risk groups in endemic countries [10], a much-needed step in the right direction to protect at-risk populations from a preventable disease, since mass vaccination is not justified against these diseases that occur in small, infrequent outbreaks [9], compared to other diseases constantly present in the population, such as measles or influenza.

Prophylactic vaccination of at-risk frontline workers against EBOV is important to minimize the spread of the virus after it is introduced into the human population. However, this should not stop the implementation of emergency ring-vaccination (vaccinating people who have been in close contact with confirmed or suspected cases) during outbreaks (Fig 1), no matter which vaccine is being used. In fact, a homologous prime/boost vaccination with VSV-EBOV, giving the second dose after18 months, has been shown in a clinical trial to be beneficial for the durability of the elicited antibody response [11]. The concept of prime vaccination followed by multiple heterologous boosts (“mix & match”) has been successfully applied for COVID-19, with mRNA, protein and viral vector vaccines being used as boosters, and may be applied to other viruses like dengue [12]. While filoviruses demonstrate much less genomic variation compared to respiratory viruses like SARS-CoV-2, it is still important to explore application of a similar strategy to all filoviruses. The approval of two vaccines for EBOV presents a starting point to explore heterologous vaccination, because it is likely that frontline workers may receive the Ad26-EBOV/MVA-BN-filo during at-risk vaccination and the VSV-EBOV as an emergency boost in case of an outbreak ring-vaccination (Fig 1). Research into this topic in animal models or human clinical trials is limited and challenging since it requires sharing of product from different developers, being poised to act when the next oubreak emerges and being able to vaccinate a sufficient number of people to obtain meaningful conclusions. However, a lot is at stake and this line of research should be emphasized in the future to select the most effective vaccination strategies.

With overlapping endemic areas of filoviruses in Africa, ongoing vaccine development efforts should ideally be expanded to identify the most effective vaccination strategies against EBOV, SUDV, MARV and ideally BDBV. This could be achieved by the development of pan-filovirus vaccines or prime/boost vaccination strategies combining virus-specific vaccines. Several vaccine platforms have been explored for a pan-filovirus vaccine, with protein subunit and VSV-based vaccines as the most promising candidates. Both platforms face their own challenges, whether it is the requirement for multi-dose administration, in the case of a subunit vaccine, or the requirement for cold-chain maintenance, in the case of a VSV-based vaccine. However, both approaches have demonstrated efficacy against lethal, high-dose challenge in NHPs against EBOV, SUDV and MARV [1]. The field still needs to define a correlate of protection—a measure to determine whether a vaccination is efficacious—applicable to all filovirus vaccines, regardless of the filovirus(es) targeted and regardless of the platform technology used. In addition, our knowledge about the durability of the immune responses elicited by these vaccines is limited and little-to-nothing is known about potential boosting strategies using virus-specific vaccines, which needs to be explored, as some may have the potential to cause immune interference.

What lies ahead for filovirus vaccine development? During previous EBOV outbreaks, EBOV-specific vaccines were successfully deployed using ring-vaccination strategies [7]. At a meeting in 2022 of stakeholders engaged in the advancement of filovirus countermeasures, including vaccines, several avenues were outlined and are currently being pursued [13], ranging from virus-specific vaccine development to pan-filovirus vaccines. Both approaches are needed to support African countries in preparation for and during filovirus outbreaks, including preventive vaccination of the at-risk population, for which a pan-filovirus vaccine seems ideal, as these viruses overlap in their endemic areas. Equally important is the implementation of ring-vaccination during outbreaks, using a vaccine specific for the outbreak-causing filovirus, under clinical trial protocols coordinated by the World Health Organization. Some of these actions have already improved outbreak response, as demonstrated in Rwanda’s swift and successful response to a MARV outbreak in 2024. A chimpanzee adenovirus vaccine was deployed shortly after the outbreak was announced, and administered in a phase 2 clinical trial to more than 1,600 people following a ring vaccination strategy [14].

Substantial improvements have been made to the disease outcomes of filovirus-infected people in Africa since the West African epidemic. Nevertheless, the knowledge gaps outlined above should be addressed with priority to improve our responses to new outbreaks. As new countermeasures against EBOV, MARV, SUDV, and other filoviruses are developed, there is hope that the diseases these viruses cause will become preventable and treatable. The expertise we have developed in combatting filovirus infection in the last decade may also serve as a roadmap to respond to other diseases that have similar public health impact.
